# Efficient compression of SARS-CoV-2 genome data using Nucleotide Archival Format

**DOI:** 10.1016/j.patter.2022.100562

**Published:** 2022-07-07

**Authors:** Kirill Kryukov, Lihua Jin, So Nakagawa

**Affiliations:** 1 Department of Informatics, National Institute of Genetics, Mishima, Shizuoka 411-8540, Japan; 2 Genomus Co., Ltd., Sagamihara, Kanagawa 252-0226, Japan; 3 Department of Molecular Life Science, Tokai University School of Medicine, Isehara, Kanagawa 259-1193, Japan

**Keywords:** SARS-CoV-2 genome database, sequence data compression, International Nucleotide Sequence Database Collaboration, INSDC, Global Initiative on Sharing Avian Flu Data, GISAID, Nucleotide Archival Format, NAF

## Abstract

Severe acute respiratory syndrome coronavirus 2 (SARS-CoV-2) genome data are essential for epidemiology, vaccine development, and tracking emerging variants. Millions of SARS-CoV-2 genomes have been sequenced during the pandemic. However, downloading SARS-CoV-2 genomes from databases is slow and unreliable, largely due to suboptimal choice of compression method. We evaluated the available compressors and found that Nucleotide Archival Format (NAF) would provide a drastic improvement compared with current methods. For Global Initiative on Sharing Avian Flu Data’s (GISAID) pre-compressed datasets, NAF would increase efficiency 52.2 times for gzip-compressed data and 3.7 times for xz-compressed data. For DNA DataBank of Japan (DDBJ), NAF would improve throughput 40 times for gzip-compressed data. For GenBank and European Nucleotide Archive (ENA), NAF would accelerate data distribution by a factor of 29.3 times compared with uncompressed FASTA. This article provides a tutorial for installing and using NAF. Offering a NAF download option in sequence databases would provide a significant saving of time, bandwidth, and disk space and accelerate biological and medical research worldwide.

## The bigger picture

Sequence data undergo explosive growth, best exemplified by the huge number of severe acute respiratory syndrome coronavirus 2 (SARS-CoV-2) genomes accumulated during just 2 years of the pandemic. To date, more than 11 million SARS-CoV-2 genomes have been sequenced and deposited in databases. Downloading and using these massive data is a challenge due to suboptimal compression used by sequence databases. In this paper, we compared the available compressors on SARS-CoV-2 genome data and found that Nucleotide Archival Format (NAF) provides a dramatic improvement to file sizes, download speeds, and decompression times. Compared with currently used methods, NAF provides up to 30–50 times better efficiency of data distribution. Timely analysis of new SARS-CoV-2 genomes is important for controlling the pandemic; therefore, it would be beneficial to use NAF for distributing these data. More generally, we believe that using NAF in sequence databases would make it easier for researchers worldwide to access molecular sequence data.

## Teaser

Vast SARS-CoV-2 genome data have accumulated in sequence databases during the pandemic. These data are currently distributed using suboptimal compression, wasting disk space, bandwidth, and time. Kryukov et al. compared 34 compressors and found that Nucleotide Archival Format (NAF) would significantly improve compactness and efficiency of sharing these data. This paper provides a detailed comparison of compressor performances and criteria for choosing an optimal compressor. It also provides a tutorial for installing and using NAF.

## Introduction

Whole-genome sequencing of severe acute respiratory syndrome coronavirus 2 (SARS-CoV-2) samples is routinely used to track the spread of the virus, understand the relationship between its strains, and develop vaccines.^1^^,^^2^ New variants that acquired novel characteristics—transmission, virulence, and therapeutic/vaccine efficacy—can be predicted based on mutation information.^3^ Therefore, timely distribution and analysis of SARS-CoV-2 genome data are critically crucial for responding to the rapid evolution of SARS-CoV-2 that potentially changes various situations around the world.

Millions of SARS-CoV-2 genomes have been already sequenced by various research groups worldwide and deposited into sequence databases. At the beginning of the coronavirus 2019 (COVID-19) pandemic, Global Initiative on Sharing Avian Flu Data^1^ (GISAID; https://www.gisaid.org/) emerged as the primary repository for exchanging SARS-CoV-2 genome data. As of June 2022, it stores more than 11 million SARS-CoV-2 genomes with severe access restrictions. Alternatively, SARS-CoV-2 genome data are also available in databases of the International Nucleotide Sequence Database Collaboration^4^ (INSDC), which includes DNA DataBank of Japan^5^ (DDBJ), European Nucleotide Archive^6^ (ENA), and GenBank.^7^ These open databases provide unrestricted access to their data and currently (as of June 2022) store more than 5 million SARS-CoV-2 genomes.

We noticed that downloading the entire set of SARS-CoV-2 genomes from these databases is often difficult. Problems include incomplete downloads, lengthy download times, and ample disk space required for processing and storing the data. We identified the root of these problems to be suboptimal choices of the data-compression methods (or no compression in the case of NCBI and ENA). Therefore, we evaluated the available data-compression methods that could be used for distributing SARS-CoV-2 data. We compared 36 compressors on SARS-CoV-2 genomes and on other repetitive datasets. We found that Nucleotide Archival Format^8^ (NAF) provides the best balance of compression strength and speed, making it the most suitable compressed format for distributing extensive repetitive sequence data.

This article is organized as follows. First, we review the current options for downloading SARS-CoV-2 data from major databases and point out their limitations. We then provide a brief overview of sequence compression. We then discuss selecting a suitable compression method for SARS-CoV-2 data. Next, we quantify the gains that can be realized by using a better compressor (NAF) for distributing SARS-CoV-2 data. We then introduce a NAF-compressed dataset consisting of all public SARS-CoV-2 sequences to date. Finally, we provide a tutorial for installing and using NAF.

## Experiences downloading SARS-2-CoV genome data

This section describes our (and a hypothetical typical user’s) experiences when attempting to download the entire set of SARS-CoV-2 nucleotide sequences from major databases. We outline the limitations and inefficiencies we encountered with each database.

### GISAID, genomic epidemiology

GISAID data are available for downloading only to registered users. After logging in to the GISAID main page, we navigated to “EpiCoV”, then “Downloads”, and in the “Genomic epidemiology” section, we selected a “FASTA” file. The downloaded file “sequences_2021-12-10_16–41.fasta.tar.gz” was 52.1 GB in size, and the decompressed “sequences.fasta” was 175 GB. The dataset contained 5,866,384 sequences. Two problems are apparent with this distribution format: (1) the extremely inefficient compression with a ratio of 3.35 times, and (2) the use of tar as an intermediate format requires decompressing the archive in two steps, consuming a total of about 402 GB of disk space. The sole purpose of using the tar package appears to include the “readme.txt” file, which describes the data-usage terms. We believe that this is a suboptimal and unnecessary choice, impacting the convenience of using the data, for a questionable benefit. The standard practice of distributing restricted datasets normally requires an agreement with the terms on the download page and then provides the usual gzip-compressed FASTA file. We note that the GISAID webpage does require agreeing with the data-use terms before showing download links. Therefore, introducing an additional “readme.txt” file in the downloadable package seems redundant.

### GISAID, download package

In the same download page in the GISAID EpiCoV website, another FASTA file exists in the “Download package” section. We downloaded it on January 18, 2022. The downloaded file “sequences_fasta_2022_01_17.tar.xz” occupied 2.31 GB and the decompressed “sequences_fasta_2022_01_17.tar” was 213 GB, and then the tar file unpacked into the “readme.txt” (830 bytes) and “sequences.fasta” (213 GB). The dataset contained 7,148,442 sequences. Using the xz compressor produced a compression ratio of 92.6—a much more efficient value than gzip, although still far from optimal. Unfortunately, this download also uses intermediate tar format to bundle the redundant “readme.txt” file. As a result, decompressing the FASTA-formatted data (in two steps) consumes 429 GB of disk space in total.

Another serious problem with this downloaded dataset is that it appears to not use unique sequence names. In one instance, as many as 8 different sequences share the same name. In total, 8,644 sequences have non-unique names in this dataset. Although GISAID assigns a unique “gisaid id” to each sequence in their database, for some reason, they do not use these unique ids in this FASTA file, making establishing a 1-to-1 correspondence between sequences and metadata impossible.

### DDBJ

From the DDBJ main page (https://www.ddbj.nig.ac.jp/index-e.html), we navigated to “Services”, then “ARSA”, where we searched for “Organism: (Severe acute respiratory syndrome coronavirus 2)”. The search returned 3,354,578 entries (on January 17, 2022). We proceeded to select “Fasta” and click “Download All”. This resulted in the downloaded file “arsa_result.fasta.gz” of 4.49 GB in size. Despite a not very large file size, downloading took several hours, probably because the data was being gzipped on the fly on the server side. The archive decompressed into a file “arsa_result.fasta” of 30.4 GB. This gives a rather inefficient compression with a ratio of 6.78 times.

Another problem is that this procedure failed to download all 3,354,578 sequences. The downloaded FASTA file contained 999,918 sequences. The download was shown as finished successfully, and the gzip archive was not malformed; the transfer occurred without errors. Combined with the nearly round number of sequences (nearly 1 million), this suggests a probable limitation on the number of sequences in a single download on the ARSA server side. It is possible to overcome this limitation by downloading multiple partial datasets using a date filter, e.g., by adding “Date:[20191201 TO 20211014]” to the search query. The user would have to be careful not to exceed the limit within each part and assemble the downloaded parts together for the complete set of genomes. A more convenient option of downloading the entire dataset as a single file seems to be not supported as of June 2022.

### ENA

From the main ENA page (https://www.ebi.ac.uk/ena/browser/home), we clicked “Search”, then “Advanced Search”. We chose “Data type:” as “Nucleotide sequences”, then clicked “Next”. We then entered the query “tax_tree (2697049)” and clicked “Search”. This resulted in 3,178,769 entries (on January 18, 2022). We then clicked on the “FASTA” link located next to the “Download ENA records:” title. This procedure resulted in the downloaded file “ena_sequence_20220118-0949.fasta” of 38.1 GB. Due to the sheer size of uncompressed data, the download took several hours. However, the downloaded file was incomplete, including only 1,251,333 sequences, which is similar to DDBJ’s case. The transfer was reported as completed successfully, and the last sequence was intact (not truncated). Therefore, the problem of the incomplete downloaded file was not due to an interrupted download but rather was caused by the server.

### GenBank

From the NCBI main page (https://www.ncbi.nlm.nih.gov/), we selected the “Nucleotide” database in the drop-down selector, then entered the query as “"Severe acute respiratory syndrome coronavirus 2"[Organism]”, then clicked “Search”. The search found 3,360,893 entries (on January 17, 2022). We proceeded to click “Sent to:”, select “File”, choose format “FASTA”, select “Sort by” as “Default order”, and click “Create File”. This resulted in downloading a file “sequence.fasta” of 102 GB. The download took more than 30 h. The downloaded file contained 3,360,674 sequences, 219 fewer than what was reported on the search result page.

### Summary

It is not easy to download the entire collection of available SARS-CoV-2 genomes from major databases. DDBJ and ENA failed to deliver the complete set of sequences as a single download after spending hours downloading the partial data. Downloading from GenBank mostly succeeded after taking more than 30 h. GISAID’s “Genomic epidemiology” FASTA dataset uses an inefficient gzip compression. GISAID’s “Download package” FASTA file contains non-uniquely named sequences and uses a suboptimal xz compression. Also, GISAID provides its FASTA-formatted sequences in an unfriendly format using intermediate tar packing, which requires a two-step unpacking before obtaining the sequence data.

GISAID still continues to lead in the number of accumulated SARS-CoV-2 genomes, storing about two times more data than the INSDC member databases. This means that it is difficult to avoid using GISAID in many areas of SARS-CoV-2 research. The problem, however, is that GISAID data are not open.^9^ Registration and approval by GISAID staff are required before accessing the content of the database. Since GISAID does not allow redistribution of their data, any inefficiencies with their data-distribution method cannot be solved by a third party via repackaging their data in a more efficient format.

### Sequence-compression overview

Timely access to the latest SARS-CoV-2 sequence data is essential for monitoring, researching, and responding to the ongoing pandemic. As we showed in the previous section, downloading large sequence datasets may take a long time and consume a lot of potentially expensive network bandwidth. It may be especially problematic in developing countries with slow internet connections. Therefore, it is natural to think about transferring the data in compressed form to enable faster downloads and reduce transfer costs. The question then becomes which of the available compression methods should be preferred.

FASTA is the established format for storing molecular sequence data. It owes its success to its simplicity and the popularity of the FASTA alignment software suite,^10^ where it was first introduced. Since FASTA is a text-based format, it can be easily manipulated, either manually or using standard and specialized software tools. Despite recent developments, such as graph-based formats (e.g., Li et al.^11^), FASTA format remains widely used in sequence databases.

A critical limitation of the FASTA format, however, is its inefficiency. FASTA encodes each nucleotide base one by one, using separate text characters. Since DNA sequences use only four nucleotide codes (with some additional codes for ambiguous cases), and text is stored using 8 bits per character, this means that a FASTA format wastes ∼75% of its size. Also, DNA often contains repeats and multiple copies of the same fragment. Finally, often multiple similar sequences are stored together in the same file, such as in the case of SARS-CoV-2 genomes. Data compression can exploit these redundancies and drastically reduce the file sizes. Indeed, today FASTA-formatted datasets are usually distributed in compressed form.

Most sequence databases currently rely on gzip for data compression. Gzip was originally released in 1992 and became popular due to being free and open source, portable, robust, having low memory overhead, and providing acceptable speed and compactness compared with alternatives at the time. These days, gzip performance is mediocre compared with the alternatives, but it remains popular because of inertia and because gzip support is integrated into many sequence-analysis tools. In addition to gzip, GISAID uses xz, another general-purpose compressor. It provides stronger compression than gzip, although it has higher computational demands during compression.

The first practical specialized compressor for sequence data was biocompress.^12^ Since then, numerous other specialized sequence compressors have been developed (see, e.g., Deorowicz and Grabowski^13^ and Hernaez et al.^14^ for review). Many early compressors, including biocompress, are not available or supported anymore, but some can still be used today, such as dnaX.^15^ Early solutions for compact storage of DNA data also included database formats for homology search tools: BLAST^16^ and BLAT.^17^ Several compressors were developed with the primary goal of providing maximum compactness: XM,^18^ DNA-COMPACT,^19^ GeCo,^20^ GeCo2,^21^ JARVIS,^22^ and GeCo3.^23^ Some closed source or non-free compressors can be possibly used in limited applications: DELIMINATE,^24^ MFCompress,^25^ ALAPY,^26^ and GTZ.^27^ Some experimental tools provide limited DNA compression: Pufferfish,^28^ UHT,^29^ and NUHT.^30^ In addition, many FASTQ compressors can be adapted for FASTA-formatted DNA data compression: beetl,^31^ DSRC,^32^ Quip,^33^ fastqz,^34^ fqzcomp,^34^ Leon,^35^ LFQC,^36^ KIC,^37^ HARC,^38^ LFastqC,^39^ Minicom,^40^ SPRING,^41^ and FQSqueezer.^42^

Specialized sequence compressors can be classified into two categories: referential and reference free. Referential methods rely on a reference genome, to which all sequences are aligned, and then only the differences are stored.^43^^,^^44^ Conversely, reference-free compressors compress just the provided data without depending on any reference. Since referential compressors always need a reference genome, they are applicable to some datasets, but not others, where such a reference is missing. Also, selecting a suitable reference genome, and distributing it together with the compressed dataset, adds substantial complexity to operating such compressors. Even though referential compression can be applied to SARS-CoV-2 sequences (e.g., Tang and Li^45^), due to the mentioned reasons, we do not consider it a viable alternative to gzip.

Additionally, recently, a new kind of specialized compressors have been developed specifically for storing collections of genomes, such as MBGC^46^ and AGC.^47^ These compressors provide good compactness, but similarly to referential compressors, they require a careful application to only suitable kinds of data. Considering that specialized compressors mostly failed to replace gzip in public databases, we realize that any prospective gzip replacement must provide as little as possible friction of switching. Such a replacement compressor must not require a reference genome, and it must be applicable to as wide as possible range of sequence data. Thus, we consider only reference-free sequence compressors to be suitable for the purpose of data distribution by sequence databases.

Previously, Liiv^48^ evaluated the performance of compressors on SARS-CoV-2 genomes. However, Liiv’s benchmark has some limitations: (1) it does not show compression time and memory consumption, (2) it includes a limited selection of relevant compressors, and (3) it includes many compressors that are only available as Windows binaries. This makes it difficult to interpret the results and select a suitable compressor for large sequence datasets.

We previously comprehensively evaluated the performance of various relevant compressors on several sequence datasets, summarized in the Sequence Compression Benchmark^49^ (SCB; http://kirr.dyndns.org/sequence-compression-benchmark/). As of June 2022, SCB includes 50 compressors (31 specialized and 19 general purpose) and a diverse set of test data. In this benchmark, in addition to compression strength, we measured the time and memory required for compression and decompression and computed several derived metrics (compression-decompression speed, transfer time and speed, transfer + decompression time and speed, compression + transfer + decompression time and speed). The test data include individual assembled genomes, collections of genomes, repetitive sequence datasets, RNA gene datasets, and protein datasets. Now, we also introduced a SARS-CoV-2 dataset into this benchmark. Therefore, SCB provides detailed data for evaluating various compressors and selecting the most suitable compressor for a given application and type of sequence data.

### Selecting compressor for SARS-CoV-2 sequence data

There are two general patterns of distributing database sequence data. (1) A prepared fixed dataset, compressed and stored on the database server, is distributed to users. Even if this dataset is updated periodically, it remains fixed between the updates. This is what GISAID does with its FASTA-format datasets. Every several days, an updated FASTA file is prepared, compressed, and shared on the GISAID website, where it stays the same until the next update. (2) Sequences are looked up in the database dynamically according to the user’s query and sent to the user, with or without compression (performed on the fly). This is how INSDC member databases operate. DDBJ compresses the sequences using gzip while sending it to the user. ENA and GenBank stream the raw uncompressed FASTA-formatted sequences.

Depending on which of these scenarios is used, different criteria become essential for selecting a compressor. For first use case, the most important measure is the time required for transferring and decompressing the data. Compression speed is less important because compression is performed only once, while decompression is performed by every user of the data. For the second case, the total time required for compression, transfer, and decompression, should be minimized. Additionally, in the first case, multi-threaded compression can be employed while compressing a fixed dataset because the entire multi-core machine can be dedicated to the task. In the second case, on the other hand, single-thread compression is usually preferable because the server is often serving multiple requests simultaneously. Thus, we consider transfer + decompression speed the critical measure for selecting a compressor for the first case and single-threaded compression + transfer + decompression for the second case. In addition to these two measures, compression strength also remains an important measure, as data compactness is important when storing large data, when copying it between machines, and when loading it into memory from disk for decompression.

**Figure 1:**
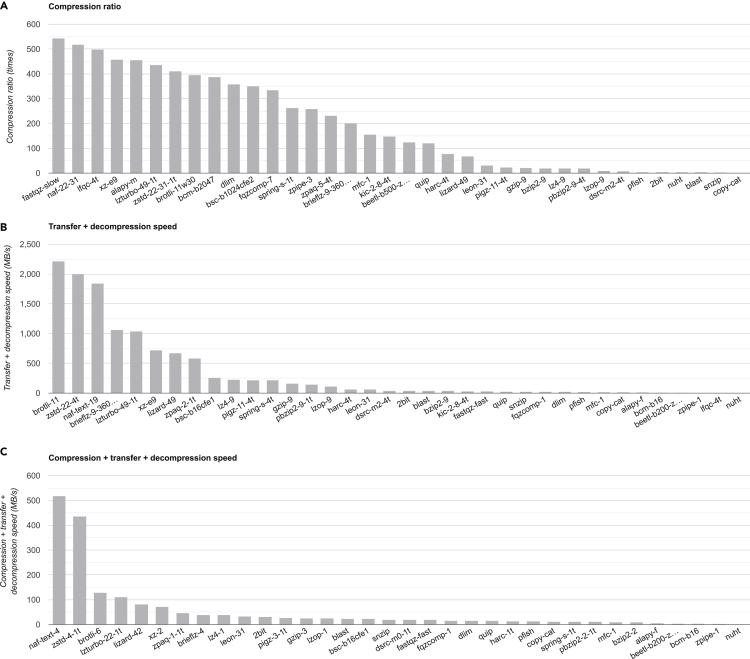
Comparison of compressors on SARS-CoV-2 genomes The test dataset is 100,000 SARS-CoV-2 genomes (3.05 GB) selected randomly from the entire set of SARS-CoV-2 genomes downloaded from GenBank on January 17, 2022, which is available in the Sequence Compression Benchmark database (http://kirr.dyndns.org/sequence-compression-benchmark/). (A–C) The best settings of each compressor are selected according to the measures compression ratio (A), transfer + decompression speed (B), and single-threaded compression + transfer + decompression speed (C). 100 Mbit/sec link speed is used for calculating transfer time.

Comparison of compressors on these measures can be visualized on the SCB benchmark website for any of the included datasets. Recently, we added a SARS-CoV-2 dataset to the benchmark. For these data, we randomly sampled 100,000 sequences (3.05 GB) of at least 25 kbp from the entire set of SARS-CoV-2 genomes downloaded from GenBank on January 17, 2022. Among the SCB test data, several other datasets exhibited high redundancy: 16S rRNA gene sequences, mitochondrion genomes, influenza genomes, human viruses, and *Helicobacter* genomes. Therefore, all these datasets should be considered for evaluating the applicability of each compressor to large, repetitive sequence data. We extracted the results of the best settings of each compressor on the SARS-CoV-2 dataset (Figure 1; Table S1) and on the remaining repetitive datasets (Figure S1). This result can also be visualized on the dynamic website of the SCB database (http://kirr.dyndns.org/sequence-compression-benchmark/).

Inspecting the benchmark results, we find that NAF^8^ provides a good balance of compactness and speed on repetitive sequence data, using the mentioned measures. On SARS-CoV-2 data, NAF is surpassed by fastqz^34^ in compression strength and by brotli^50^ and zstd^51^ in transfer + decompression speed on the SARS-CoV-2 data, and it leads in single-threaded compressions + transfer + decompression speed. On other repetitive datasets, NAF leads in all three metrics.

An important quality of a compressor is compatibility with different kinds of data and usage scenarios. General-purpose compressors, such gzip, support a maximally broad range of uses. Therefore, any potential replacement must also strive to be useful in diverse scenarios. NAF has a comprehensive feature set in this regard, supporting both FASTA and FASTQ formats and supporting not only DNA but also RNA and protein data. For DNA/RNA data, NAF supports ambiguous nucleotide codes and upper/lowercase masking. Also, NAF can compress alignments containing gaps denoted as the “-” character. Strong performance benchmark results, and applicability to a wide range of data, allow us to recommend NAF for the general application of distributing large sequence datasets.

## Compressing SARS-CoV-2 sequence data

We then quantified performance gains that can be achieved by applying a more efficient compressor (i.e., NAF) to the distribution of SARS-CoV-2 data. The current approaches are sending uncompressed FASTA data by ENA and GenBank, compressing on the fly with gzip by DDBJ, and pre-compressing with gzip and xz by GISAID. Thus, we measured the performance of NAF, gzip, and xz on SARS-CoV-2 genome data. We used the GISAID “Genomic epidemiology FASTA-formatted dataset as the test data (175 GB, 5,866,384 sequences). We compared gzip 1.11, xz 5.2.5, and NAF 1.3.0, by using the representative range of settings of these compressors: 1–9 for gzip and xz, and 1–22 for NAF. When compressing and decompressing, we loaded the entire input into the disk cache first (using “cat >/dev/null”). During decompression, the output was piped to /dev/null to avoid spending time and to save disk space. We timed compression and decompression and measured memory consumption using GNU Time (“/usr/bin/time -v”). Same with the SCB, we used the link speed of 100 Mbit/sec for calculating transfer time since this is the standard speed of a broadband internet connection.

**Figure 2:**
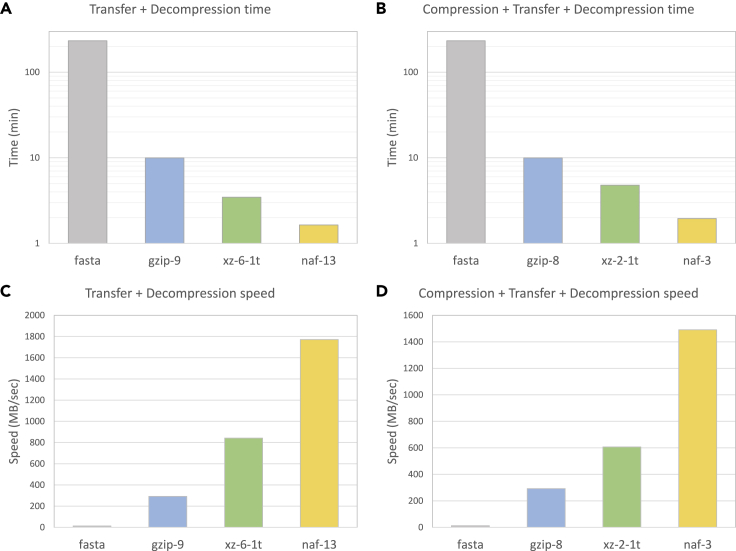
Comparison of best-performing settings of gzip, xz, and naf (A and B) Performance in terms of time (on a log scale) required to complete transfer + decompression (A) and compression + transfer + decompression (B). (C and D) The same results but in terms of speed (in MB/s), computed as uncompressed data size divided by the time required for transfer + decompression (C) and compression + transfer + decompression (D).

The benchmark results for this dataset are shown in Table S2 and Figure S2. We then selected the best settings of each compressor based on the two measures discussed above. The benchmark results of these selected settings are shown in Table S3 and Figure 2. These results allow us to quantify the efficiency improvement that would result from adding NAF as an optional format for distributing SARS-CoV-2 genome datasets.

For distributing a static SARS-CoV-2 genome dataset (e.g., for GISAID use case), we are interested in the shortest transfer + decompression time. In this scenario, using NAF reduces transfer + decompression time 141.7 times compared with distributing an uncompressed FASTA file, 6.1 times compared with using gzip, and 2.1 times compared with using xz compression (using the best settings of gzip and xz).

Compared with the gzip settings actually used by GISAID, NAF would provide 52.2 times improvement (naf-13 compared with gzip-4). It is not clear what xz setting is used by GISAID. The compression ratio of their xz-compressed data is about 92.2 times, which would place it somewhere between levels 1 and 2. Using this compression ratio and decompression speed of xz-2, we would get a transfer + decompression speed of 473 MB/s, which is 3.7 times slower than naf-13’s speed of 1,772 MB/s.

For sending a dynamically selected set of sequences (such as what is done by DDBJ, GenBank, and ENA), we are interested in compression + transfer + decompression time. For this case, using NAF reduces the total time required for the three steps 29.3 times compared with sending uncompressed FASTA (such as what is done by GenBank and ENA), 14.5 times compared with using gzip compression (used by DDBJ), and 3.4 times compared with using xz compression. We observed a compression ratio of 6.78 times in the DDBJ file, which is closest to the gzip-6 result in our test. gzip-6 provides a 9.15 MB/s compression + transfer + decompression throughput in our analysis, which is 40 times slower than 366.61 MB/s provided by NAF (naf-3). For storing SARS-CoV-2 genome data, using the strongest setting of each compressor, NAF provides 8.33 times stronger compression than gzip and 1.09 times stronger compression than xz.

### NAF-compressed SARS-CoV-2 dataset

In order to provide a practical demonstration of the sequence data distribution strategy outlined in this paper, we prepared a NAF-compressed dataset consisting of all public SARS-CoV-2 nucleotide sequences (up to June 17, 2022).

To prepare this dataset, we downloaded all available SARS-CoV-2 sequences from GenBank on July 17, 2022. We navigated to https://www.ncbi.nlm.nih.gov/labs/virus/, where we clicked “Search by virus”, then “Up-to-date SARS-CoV-2”. The search returned 5,588,048 sequences. We then clicked “Download”, selected “Nucleotide” in the “Sequence data (FASTA Format)” column, then clicked “Next”, selected “Download All Records”, clicked “Next”, selected “Use default” (FASTA definition line), then clicked “Download”. This resulted in downloading a 170 GB FASTA file. We compressed it into the NAF format using the command “ennaf −22 --fasta --dna -o SARS-CoV-2-NCBI-2022-06-17.naf sequences.fasta”. The compressed file occupied 251 MB.

We now make this dataset available online for the benefit of those who may need to use these data and to demonstrate the utility of the NAF format. The dataset is available at the following URL: http://sayer.nig.ac.jp/kirill/SARS-CoV-2/sequence-data/.

### NAF tutorial

Here, we describe how to apply NAF compression to your own SARS-CoV-2 genome data. We use Linux OS for this tutorial because Linux is commonly used for large-scale data analyses. However, NAF can also be used in Windows (via cygwin or WSL2) as well as MacOS.

Normally, NAF is compiled from source code. Some pre-requisites are required to compile it: git, gcc, and make. Additionally, diff and perl are used by the optional test suite. These prerequisites can be installed on Ubuntu with the following command:

sudo apt install git gcc make diffutils perl

On MacOS, they can be installed as part of the “Xcode Command Line Tools” package. With pre-requisites in place, NAF can be installed with the following commands:

git clone --recurse-submoduleshttps://github.com/KirillKryukov/naf.git
cd naf && make && make test && sudo make install

The first of these commands downloads the NAF sources. The second one builds and tests the NAF binaries “ennaf” and “unnaf” and copies them to the “/usr/local/bin” directory. An alternative location can be specified by adding “prefix = DIR” to the “make install” command. For example, without superuser permissions, NAF can be installed into the user’s home directory with “make prefix = ∼ install”. It’s also possible to omit the “make install” step and just copy the “ennaf” and “unnaf” binaries to a preferred location.

Alternatively, NAF can be also installed using Bioconda^52^ (https://bioconda.github.io/). To install Bioconda, run the following commands for MacOS:

curl -O https://repo.anaconda.com/miniconda/Miniconda3-latest-MacOSX-x86_64.sh
sh Miniconda3-latest-MacOSX-x86_64.sh

For Linux or Windows WSL2, run the following:

curl -O https://repo.anaconda.com/miniconda/Miniconda3-latest-Linux-x86_64.sh
sh Miniconda3-latest-Linux-x86_64.sh

Add the following channels including bioconda:

conda config --add channels defaults
conda config --add channels bioconda
conda config --add channels conda-forge

Then, you can install NAF via the following command:

conda install naf

After installation is complete, it can be tested by running the commands “ennaf --version” and “unnaf --version”. Then, the “ennaf” command can be used for compressing and “unnaf” for decompressing sequence data. Command-line options of these commands can be seen by running “ennaf -h” and “unnaf -h”. Simple compression of a FASTA file with default parameters can be done using this command: “ennaf file.fa -o file.naf”.

Compression level (strength) can be specified with the option “-#”, where # is the compression level from 1 to 22. 1 is the fastest and weakest level, and 22 is the slowest and strongest one. The default compression level is 1. The appropriate level can be selected based on the purpose of the compression. For a one-time transfer, level 1 may provide the best speed. For long-term storage, level 22 may be preferable, even though it takes longer to perform the compression. Level 22 is also optimal for distribution of fixed datasets by sequence databases because, in this case, the compression has to be done only once, while better compactness of the data will benefit many users.

By default, ennaf assumes that the input contains DNA sequences in FASTA format. FASTQ data can be compressed by adding “--fastq” to the compression command. RNA and amino acid sequences can be compressed using “--rna” and “--protein”, respectively. These options are not needed for the decompression step, as the decompressed data automatically matches the compressed format and sequence type.

Decompressing a NAF file can be done using “unnaf file.naf -o file.fa”. Streaming a decompressed output into the next command is the crucial use case of NAF and can be done as “unnaf file.naf >file.fa”. A FASTQ dataset compressed into NAF can be decompressed directly into FASTA format by adding the “--fasta” option to the decompression command.

Compression can be done using IO redirection: “ennaf -c <file.fa >file.naf”. This allows, for example, converting gzip-compressed data into NAF without saving the decompressed sequences, using this command: “gzip -dc file.gz | ennaf -o file.naf”. Decompressing using IO redirection is also possible and is one of the most important uses of the NAF-compressed data: “unnaf file.naf | …”.

The NAF compression process may use disk storage for temporary data. The directory for temporary data can be specified with the “--temp-dir” command line option. By default, the directory configured in the TMPDIR or TMP environment variables is used. Decompression never uses disk storage.

By default, NAF preserves the line lengths during compression and decompression. Line length can be overridden using the “--line-length N” option during either compression or decompression (applicable only to FASTA data, not FASTQ). A line length of 0 means unlimited lines, i.e., each sequence printed in a single line.

NAF compression and decompression are always single threaded. Therefore, multiple compression or decompression tasks may be executed in parallel on a multi-core machine. When designing a web server that provides dynamically compressed NAF data, care has to be taken to ensure that the total number of simultaneous compression tasks does not exceed the number of available cores (or threads for hyper-thread CPUs).

The number of potential compression tasks and the amount of available RAM have to be taken into account when choosing the NAF compression level. Level 22 may use up to about 4 GB of RAM when compressing large data, while level 1 will use about 10–15 MB. For example, for running 32 compression tasks on a 32-thread machine with 32 GB of RAM, the NAF compression level 19 can be selected because it consumes less than 500 MB or RAM, thus 32 parallel tasks may use less than half of the available RAM.

More details about using NAF are available on these GitHub pages: https://github.com/KirillKryukov/naf, https://github.com/KirillKryukov/naf/blob/master/Compress.md, and https://github.com/KirillKryukov/naf/blob/master/Decompress.md.

## Discussion and conclusion

Our results demonstrate that the recent massive SARS-CoV-2 genome data can be efficiently compressed by using NAF. Significant gains in efficiency can be unlocked by applying NAF to the distribution of SARS-CoV-2 datasets. This applies to both each user and the data-distributing center. In particular, ENA and GenBank distribute data as uncompressed FASTA files of SARS-CoV-2 genomes where NAF compression would decrease the required storage space and network bandwidth by a factor of over 500. Depending on connection speed, it would also drastically decrease download times. DDBJ uses gzip for compressing sequence data. Here, NAF would provide a 14.5 times decrease in waiting time (total time taken by compression + transfer + decompression). GISAID uses gzip and xz for pre-compressing their FASTA datasets. In this case, using NAF would provide 52.2 and 3.7 times faster downloads (compared with the gzip and xz settings used by GISAID) and 6 and 2 times faster downloads compared with the best settings of gzip and xz, respectively (for the strongest gzip setting, the one used by GISAID is even less efficient).

The best setting of each compressor may be different depending on the performance measure used as a selection criterion. Often achieving a good result in speed requires sacrificing compactness, and vice versa. Different performance measures have to be considered in combination when selecting a compressor and its settings. For example, GISAID uses gzip for distributing a static SARS-CoV-2 dataset. When considering transfer + decompression speed, level 9 outperforms other gzip levels while providing a compression ratio of 76.1 times. However, GISAID probably uses gzip-4, with much weaker compression (3.4 times), possibly saving compression time and delivering the updated dataset faster. The overall balanced setting must perform well on all metrics.

NAF compression levels between 9 and 12 can be considered balanced settings for distributing SARS-CoV-2 genome data. These settings provide compression ratios of 516.9–522.3 times, compression speeds of 360–284 MB/s, and decompression speeds around 2.2 GB/s.

For open-access databases, such as DDBJ, ENA and GenBank, it is possible to download the sequences, re-compress them into a more efficient format, and redistribute them. However, GISAID’s data-usage terms explicitly disallow redistribution. Also, recompressing and redistributing the data would consume precious time, which can be critical when accessing the latest SARS-CoV-2 genome data. Therefore, it would be much more efficient to be able to apply better compression at the source database, which would benefit all its users.

After being downloaded, sequence data are often distributed to multiple computers and used for automated analysis. In these scenarios, it is efficient to store the data in a compressed form that allows fast decompression. NAF’s high decompression speed of ∼2.2 GB/s makes it particularly suitable for such uses. gzip and xz can still be used similarly, although with much slower decompression speeds of 422 and 951 MB/s, respectively. However, GISAID’s use of an intermediate tar package is incompatible with direct access to the compressed data and requires a complete decompression before using the data.

SARS-CoV-2 genome data have been accumulating rapidly and are a crucial resource for dealing with the pandemic. Introducing a more efficient compression, such as NAF, for distributing SARS-CoV-2 genome data will allow faster detection and reaction to the emergence of new dangerous strains. It will improve the efficiency of monitoring and controlling the pandemic. Indeed, changes to various large-scale COVID-19-related data including epidemiological data are being discussed for efficient analyses (Xu et al.^53^; Kraemer et al.^54^). Therefore, to avoid disrupting the established genomic data analysis pipelines, it would be also preferable to add NAF as an optional format for downloading data rather than as the only available option.

## Supplemental information

Document S2. Tables S1–S3

Document S3. Article plus supplemental information
