# Supplementary material for: Efficient compression of SARS-CoV-2 genome data using Nucleotide Archival Format
Source: Patterns (N Y). 2022 Jul 7;3(9):100562. doi: 10.1016/j.patter.2022.100562 (PMC9259476; doi:10.1016/j.patter.2022.100562)
Supplement: Document S1. Figures S1 and S2 [file mmc1.pdf]

Patterns, Volume *3*

Supplemental information

Efficient compression of SARS-CoV-2 genome data using Nucleotide Archival Format

Kirill Kryukov, Lihua Jin, and So Nakagawa

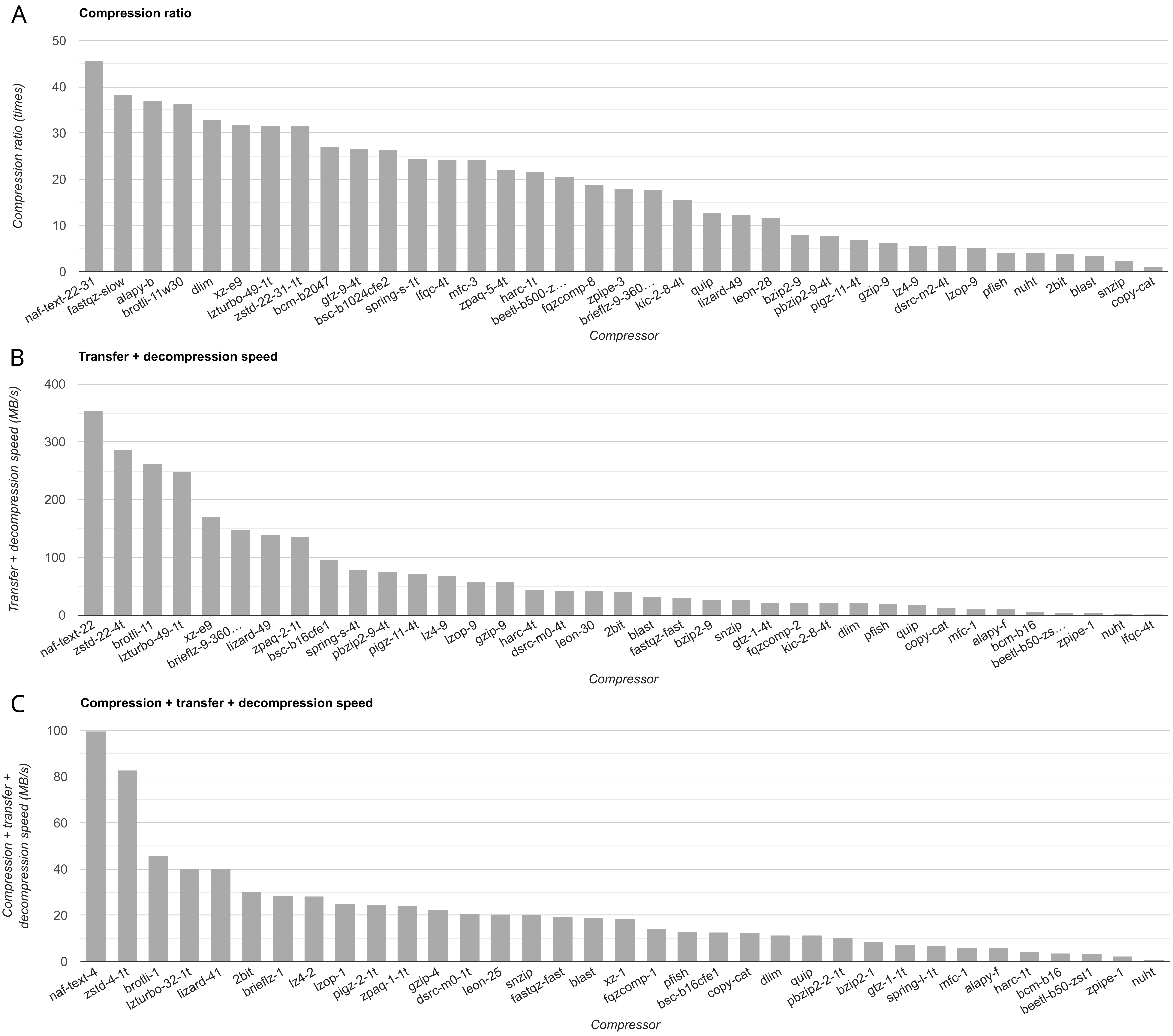

Fig. S1. Comparison of compressors on repetitive data other than SARS-CoV-2 genomes, from Sequence Compression Benchmark. Best settings are selected according to the measures shown: Compression ratio (A), Transfer + Decompression speed (B), and single threaded Compression + Transfer + Decompression speed (C). 100 Mbit/sec link speed is used for calculating transfer time. Results from multiple datasets are averaged.

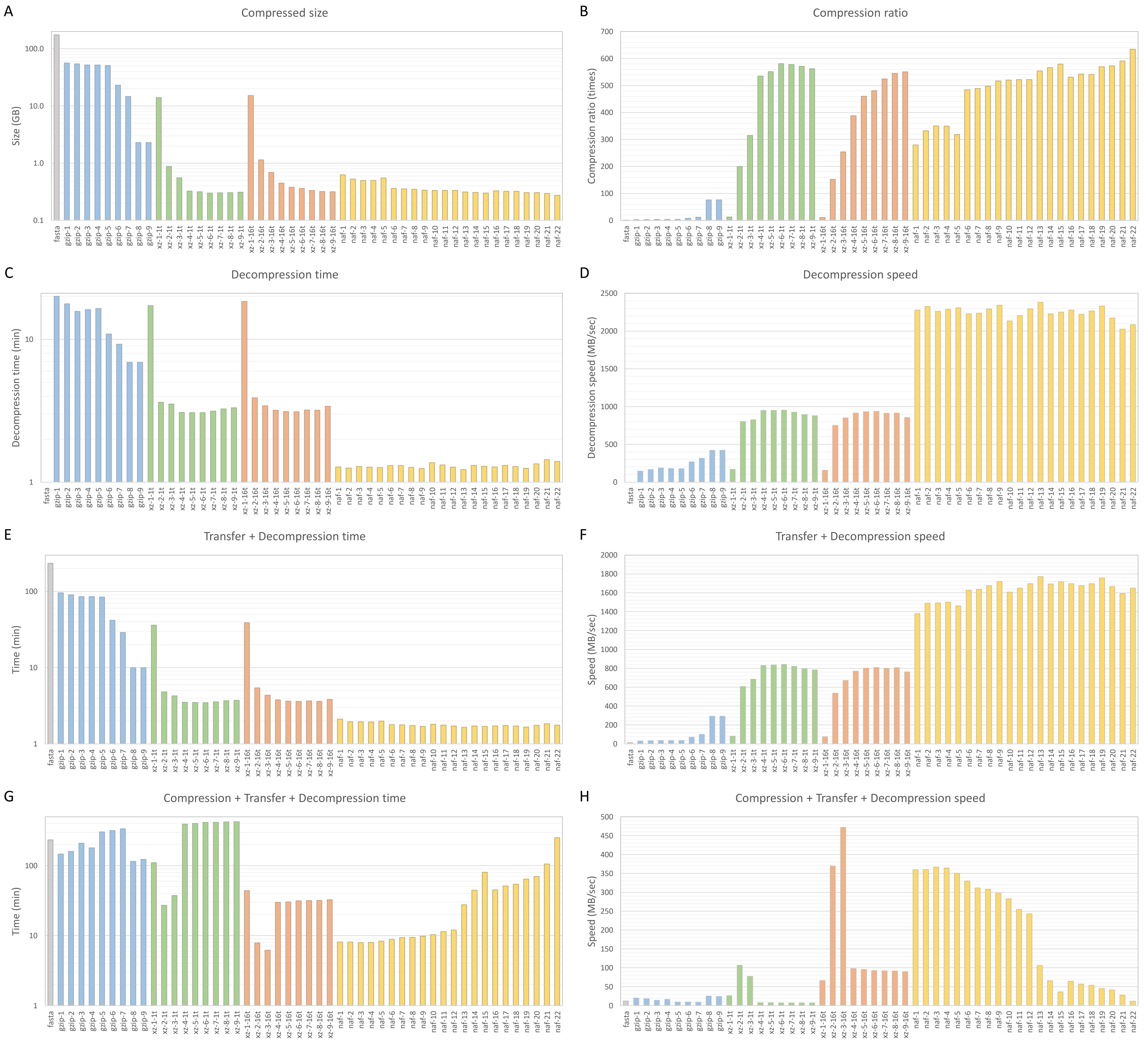

Fig. S2. Comparison of gzip, xz and naf, on the GISAID Genomic epidemiology FASTA dataset from 2021-12-10. Link speed of 100 Mbit/sec was used for calculating transfer time.
